# Prevalence and risk of *Plasmodium vivax* infection among Duffy-negative individuals: a systematic review and meta-analysis

**DOI:** 10.1038/s41598-022-07711-5

**Published:** 2022-03-07

**Authors:** Polrat Wilairatana, Frederick Ramirez Masangkay, Kwuntida Uthaisar Kotepui, Giovanni De Jesus Milanez, Manas Kotepui

**Affiliations:** 1 Department of Clinical Tropical Medicine, Faculty of Tropical Medicine, Mahidol University, Bangkok, Thailand; 2 Department of Medical Technology, Faculty of Pharmacy, University of Santo Tomas, Manila, Philippines; 3 Medical Technology, School of Allied Health Sciences, Walailak University, Tha Sala, Nakhon Si Thammarat, Thailand

**Keywords:** Parasitology, Malaria

## Abstract

A better understanding of the occurrence and risk of *Plasmodium vivax* infection among Duffy-negative individuals is required to guide further research on these infections across Africa. To address this, we used a meta-analysis approach to investigate the prevalence of *P. vivax* infection among Duffy-negative individuals and assessed the risk of infection in these individuals when compared with Duffy-positive individuals. This study was registered with The International Prospective Register of Systematic Reviews website (ID: CRD42021240202) and followed Preferred Reporting Items for Systematic review and Meta-Analyses guidelines. Literature searches were conducted using medical subject headings to retrieve relevant studies in Medline, Web of Science, and Scopus, from February 22, 2021 to January 31, 2022. Selected studies were methodologically evaluated using the Joanna Briggs Institute (JBI) Critical Appraisal Tools to assess the quality of cross-sectional, case–control, and cohort studies. The pooled prevalence of *P. vivax* infection among Duffy-negative individuals and the odds ratio (OR) of infection among these individuals when compared with Duffy-positive individuals was estimated using a random-effects model. Results from individual studies were represented in forest plots. Heterogeneity among studies was assessed using Cochrane Q and I^2^ statistics. We also performed subgroup analysis of patient demographics and other relevant variables. Publication bias among studies was assessed using funnel plot asymmetry and the Egger’s test. Of 1593 retrieved articles, 27 met eligibility criteria and were included for analysis. Of these, 24 (88.9%) reported *P. vivax* infection among Duffy-negative individuals in Africa, including Cameroon, Ethiopia, Sudan, Botswana, Nigeria, Madagascar, Angola, Benin, Kenya, Mali, Mauritania, Democratic Republic of the Congo, and Senegal; while three reported occurrences in South America (Brazil) and Asia (Iran). Among studies, 11 reported that all *P. vivax* infection cases occurred in Duffy-negative individuals (100%). Also, a meta-analysis on 14 studies showed that the pooled prevalence of *P. vivax* infection among Duffy-negative individuals was 25% (95% confidence interval (CI) − 3%–53%, I^2^ = 99.96%). A meta-analysis of 11 studies demonstrated a decreased odds of *P. vivax* infection among Duffy-negative individuals (p = 0.009, pooled OR 0.46, 95% CI 0.26–0.82, I^2^ = 80.8%). We confirmed that *P. vivax* infected Duffy-negative individuals over a wide prevalence range from 0 to 100% depending on geographical area. Future investigations on *P. vivax* infection in these individuals must determine if Duffy-negativity remains a protective factor for *P. vivax* infection.

## Introduction

While *Plasmodium falciparum* is the most prevalent malaria parasite in the World Health Organization African Region and accounted for 99.7% of estimated malaria cases in 2018^1^, there are increasing reports of *P. vivax* infection across Africa^2,3^. *P. vivax* infection of human erythrocytes requires the presence of a glycoprotein on the surface of red bloods, the Duffy blood group antigen or the Duffy Antigen Receptor for Chemokines (DARC)^4,5^. DARC is also the receptor for the simian malarial parasite, *Plasmodium knowlesi*^6^. DARC binds to *P. vivax* Duffy binding protein (PvDBP) before it invade erythrocytes^7,8^. The Duffy blood group is expressed by the *FY* gene on chromosome 1, and is genotyped as FY (a), FY (b), FY (a)^ES^, and FY (b)^ES^^9^. Duffy phenotypes, including Fy(a + b +), Fy(a + b −), and Fy(a − b +) are Duffy-positive phenotypes, while Fy(a − b −) or FY (a)^ES^(b)^ES^ are Duffy-negative phenotypes. The Fy(a − b −) phenotype is caused by homozygosity of the *FY* allele carrying a point mutation at 67T > C (rs2814778) which prevents Duffy antigen expression in red blood cells^10^.

The Duffy-negative phenotype is highly predominant in sub-Saharan African populations, with high phenotype median frequencies of 98%–100% in west, mid, and south-eastern regions^5^. Recent studies reported that Duffy-negative individuals have a risk of *P. vivax* infection^11,12^. It was also postulated that *P. vivax* infections were passed back and forth between Duffy-positive and Duffy-negative individuals by *P. vivax*-infected mosquitoes parasitizing Duffy-positive individuals and transmitting parasites to Duffy-negative individuals^13^. As *P. vivax* infection can lead to severe malaria with poor outcomes^14^, a better understanding of *P. vivax* infection occurrence and risk among Duffy-negative individuals is required to guide further epidemiological research in Africa. Therefore, using a meta-analysis approach, we investigated *P. vivax* infection prevalence among Duffy-negative individuals and assessed the risk of infection among these individuals when compared with Duffy-positive individuals.

## Methods

### Protocol and registration

This study followed Preferred Reporting Items for Systematic review and Meta-Analyses guidelines^15^. The review was registered at The International Prospective Register of Systematic Reviews website (ID: CRD42021240202).

### Search strategy

Literature searches were conducted using medical subject headings in the National Library of Medicine and terms related to *P. vivax* malaria and Duffy status. The following search terms were used: “DBP” OR “D binding protein” OR “D-element-binding protein” OR “DBP transcription factor” OR “D-site binding protein.” Search terms are shown (Table S1). Medline, Web of Science, and Scopus were searched from the February 22, 2021 to the January 31, 2022. Additional searches of reference lists and review articles were also performed to ensure literature saturation.

### Eligibility criteria

Cross-sectional, cohort, and case–control studies were considered if they reported *P. vivax* infections among Duffy-negative individuals. *P. vivax* infection was confirmed by microscopic or molecular analysis. Duffy genotypes or phenotypes were characterized by polymerase chain reaction-restriction fragment length polymorphisms, with and without sequencing. Only articles in English were included. The following articles were excluded: no Duffy-negative individuals among *P. vivax* cases, genetic analysis of the Duffy protein, no report on Duffy status, case reports and case series, experimental studies, clinical trials, and studies from which data could not be extracted.

### Study selection

Study selection was performed in Endnote (Version X8, Clarivate Analytics, USA) by two authors (PW and MK). Discrepancies between authors on study selection were resolved by consensus and discussion with a third author (KUK). After retrieving articles, duplicated articles were removed. The remaining articles were title and abstract screened, after which irrelevant studies were excluded. The remaining article texts were examined according to eligibility criteria. All excluded articles were assigned appropriate reasons. Selected articles were further extracted using a standardized pilot datasheet.

### Data extraction

The standardized pilot datasheet included the following: first author name, year of publication, study site, year the study was conducted, participants, age, gender, number of patients with malaria, number of *P. vivax* cases, number of *P. vivax* infections among Duffy-negative individuals, number of *P. vivax* infections among Duffy-positive individuals, malaria identification methods, and Duffy status. Two authors (PW and MK) independently collected these data. Disagreements over data extraction were resolved by discussion. Data were randomly checked by a third author (FRM) for completeness, plausibility, and integrity, before data was processed.

### Study quality

The methodological quality of selected studies was evaluated using the Joanna Briggs Institute (JBI) Critical Appraisal Tools for assessing cross-sectional, case–control, and cohort studies^16^. The tool for cross-sectional studies comprised eight checklists, whereas 10 and 11 were used for case–control and cohort studies, respectively. Studies with > 75%, 50%, and ≤ 50% scores indicated high, moderate, or low quality, respectively. Study quality was assessed by two authors (PW and MK).

### Study outcomes

The primary study outcome was the pooled prevalence of *P. vivax* infection among Duffy-negative individuals. The secondary outcome was the odds ratio (OR) and 95% confidence interval (CI) of *P. vivax* infection among Duffy-negative individuals when compared with Duffy-positive individuals.

### Data processing

Primary and secondary study outcomes were both estimated using the random-effects model. This model was used in the presence of heterogeneity of the effect estimates (ES) (pooled prevalence or OR); meanwhile, the fixed-effects model was used in the absence of heterogeneity of the ES. The results from individual studies were graphically represented on forest plots. Heterogeneity among studies was assessed using Cochrane Q and I^2^ statistics. A Cochrane Q p < 0.05 indicated significant heterogeneity among studies. I^2^ statistics were used to quantify heterogeneity; I^2^ > 50% indicated substantial heterogeneity. If heterogeneity existed, the random-effects model was used for pooled the pooled prevalence and OR, and if no heterogeneity was observed, the fixed-effects model was used for pooled the pooled prevalence and OR. Meta-regression analysis was performed to determine the source(s) of heterogeneity of ES (pooled prevalence, OR) among studies. If the source(s) of heterogeneity was identified, a subgroup analysis was conducted. We performed sensitivity analysis of the pooled prevalence and the odds of infection between Duffy-negative individuals using the fixed-effects model to determine the robustness of our meta-analysis results.

### Publication bias

Publication bias was assessed by visualizing funnel plot asymmetry and the Egger’s test. Funnel plot asymmetry indicated publication bias. A significant Egger’s test (p < 0.05) indicated that funnel plot asymmetry was due to a small study effect. If the funnel plot was asymmetrical (by visualization or a significant Egger’s test), a contour-enhanced funnel plot was generated to identify if funnel plot asymmetry was due to publication bias or other causes.

## Results

### Search results

**Figure 1:**
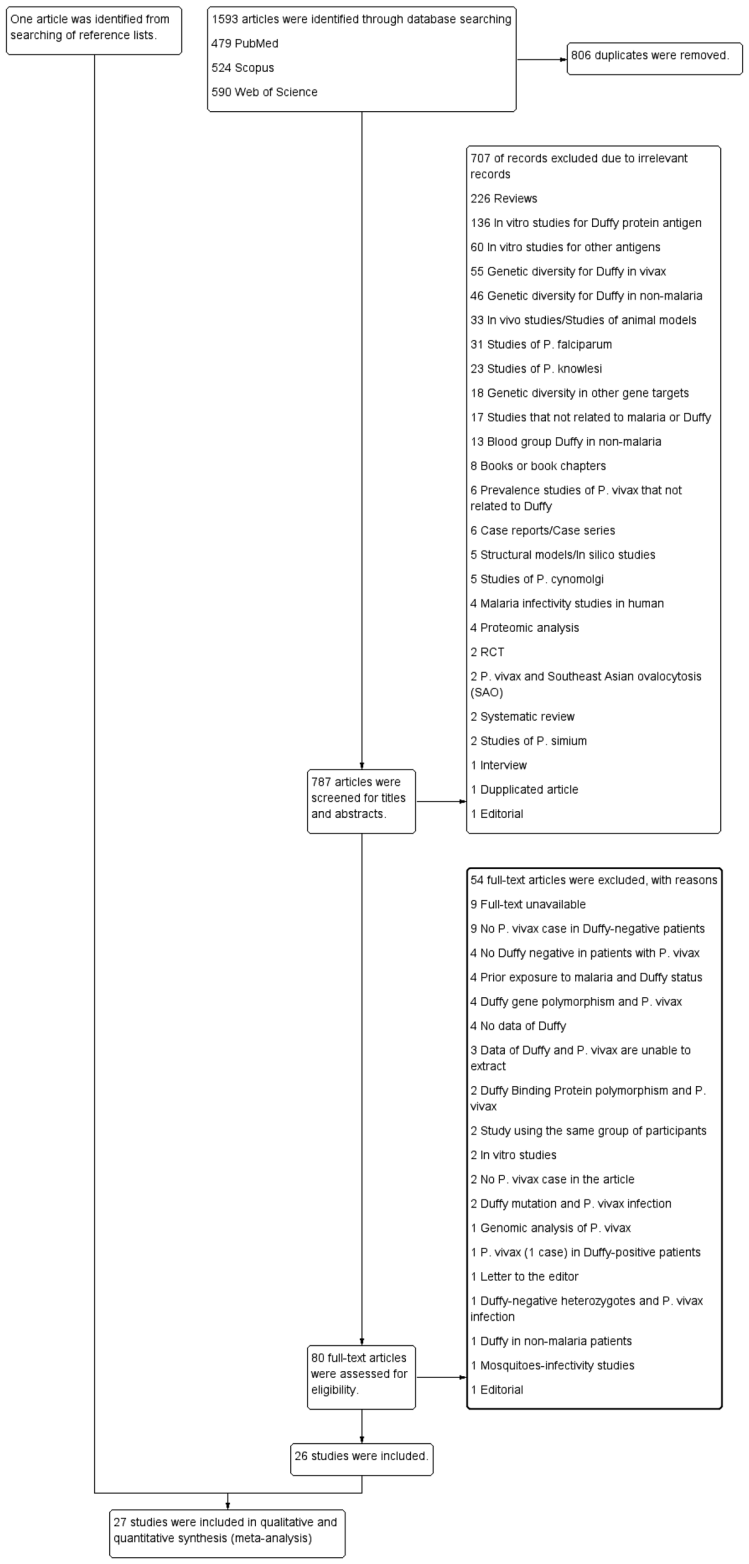
Study flow diagram demonstrating study selection process.

Of 1593 retrieved articles, 806 were retained after duplicated articles were removed. After screening title and abstracts of 787 articles, 707 were excluded due to irrelevance (Fig. 1). Thus, 80 articles were examined for full texts and 54 excluded due to the following reasons: nine full texts were unavailable, nine texts reported no *P. vivax* cases in Duffy-negative patients, four texts had no Duffy-negative patients with *P. vivax* infection, four texts indicated prior exposure to malaria and Duffy status, four texts reported Duffy gene polymorphisms and *P. vivax* infection, four texts had no Duffy data, three texts had Duffy and *P. vivax* data which could not be extracted, two reported DBP polymorphisms and *P. vivax* infection, two texts used the same participants, two texts were in vitro studies, two had no *P. vivax* cases, two reported a Duffy mutation and *P. vivax* infection, one text was a *P. vivax* genomic analysis, one text reported *P. vivax* (1 case) in Duffy-positive patients, one was a letter to the editor, one reported Duffy-negative heterozygotes and a *P. vivax* infection, one reported Duffy status in non-malaria patients, one was a mosquito-infectivity study, and one was an editorial. Thus, 26 studies^17–42^ met eligibility criteria, however, one study^43^ was identified from the bibliography of a study, therefore 27 studies^17–43^ met eligibility criteria and were included.

### Study characteristics

**Figure 2:**
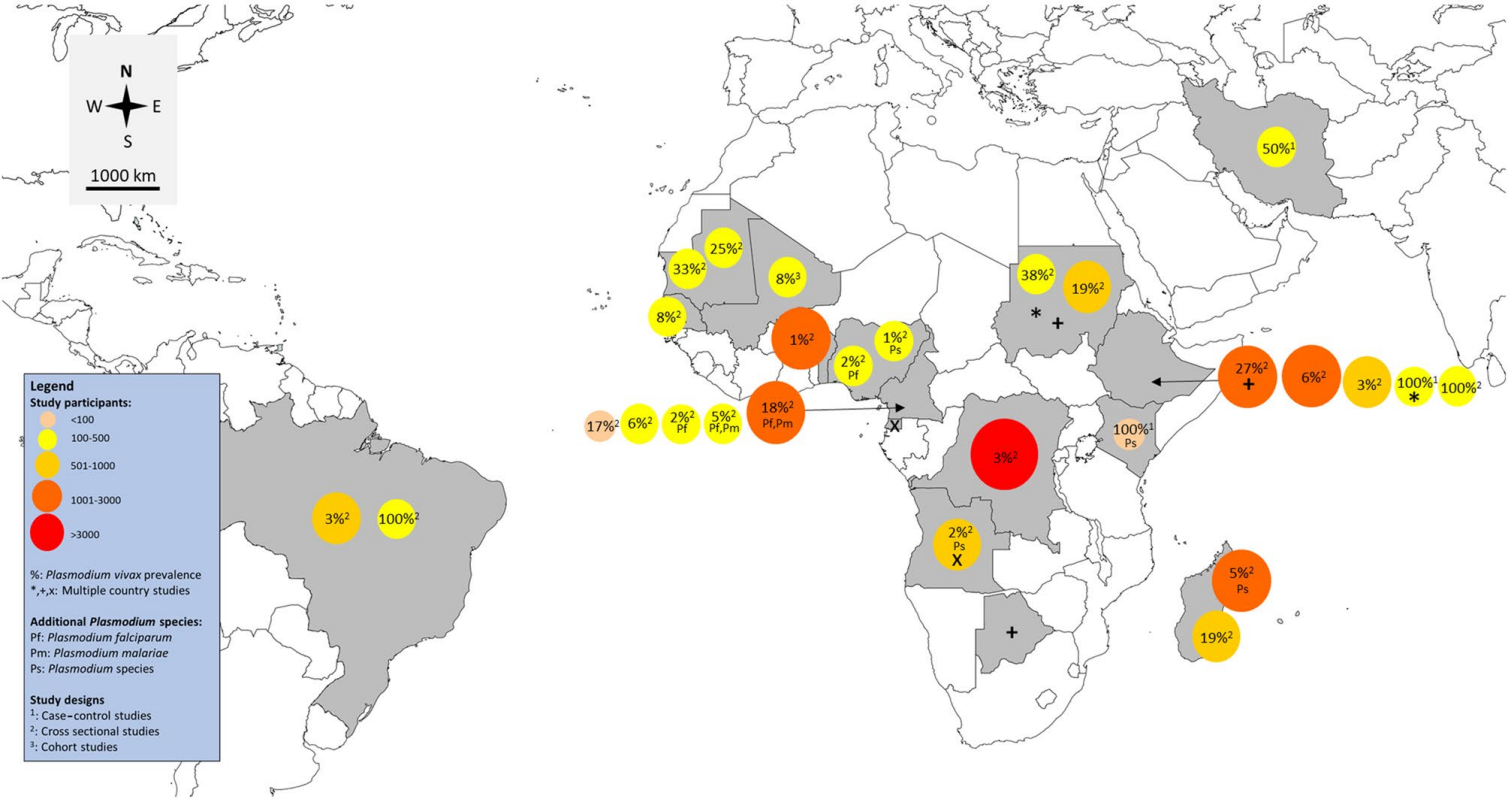
Distribution of included studies on *P. vivax* infection among Duffy-negative individuals. Map was sourced and modified from https://mapchart.net/world.html by authors. Authors were allowed to use, edit and modify any map created with mapchart.net for publication freely by adding the reference to mapchart.net in publication.

Study characteristics are shown (Table 1). All were published between 2006–2021 and almost all (24/27, 88.9%) reported *P. vivax* infection among Duffy-negative individuals in Africa. Three studies^20,21,31^ were conducted in South America (2/27, 7.4%) and Asia (1/27, 3.7%). Of the 24 African studies, six were conducted in East Africa (Ethiopia^28,43^, Madagascar^25,29^, Kenya^40^, and Ethiopia^41^), seven in Mid Africa (Democratic Republic of Congo^19^, Cameroon^22,23,32,33,39^, Angola, and Equatorial Guinea^30^), seven in West Africa (Mauritania^24,42^, Nigeria^36,37^, Senegal^34^, Mali^35^, and Benin^38^), two in North Africa (Sudan^17,18^), one in North and East Africa (Ethiopia and Sudan^26^), and one in Ethiopia/Botswana/Sudan^27^. Twenty-two articles were cross-sectional studies (22/27, 81.5%), two were case-controls^12,40^, and one was a cohort study^35^. The geographical distribution of studies is shown (Fig. 2).

**Table 1:** Characteristics of the included studies.

No	Author, year	Study area (years of the survey)	Study design	Age range (years)	Gender (male, %)	Participants	Method for *Plasmodium* spp. identification	Target gene for PCR	Number of *P. vivax* (malaria positive)	Method for Duffy antigen genotyping	Duffy status among *P. vivax* cases
1	Abdelraheem et al. (2016)	Sudan (2009)	Cross-sectional study	< 10 (38), 10–20 (9), > 20 (1)	22, 45.8	126 suspected malaria patients	Microscopy, RDT and PCR	*SSU rRNA*	48	PCR–RFLP	Duffy negative: 4/4 Duffy positive: 44
2	Albsheer et al. (2019)	Sudan (2013–2017)	Cross-sectional study	Mean 25 years	Male/female: 1.73	992 microscopy positive samples	Microscopy, PCR	*SSU rRNA*	190 (992)	Sequencing (190)	Duffy negative: 34/77 Duffy positive: 156/178
3	Brazeau et al. (2021)	Democratic Republic of the Congo (2013–2014)	Cross-sectional study	15–59 years and 15–49 years	NS	17,972 screened for *P. vivax* infection	PCR	*SSU rRNA*	467 (5646)	High-Resolution Melt (HRM)	Duffy negative: 464/467 Duffy positive: 3
4	Carvalho et al. (2012)	Brazil (2009)	Cross-sectional study	NS	NS	678 individuals	Microscopy, PCR	mtDNA	19 (137)	Sequencing	Duffy negative: 2/29 Duffy positive: 96/553
5	Cavasini et al. (2007)	Brazil (2003–2005)	Cross-sectional study	18 years	NS	312 patients with *P. vivax* infection	Microscopy, PCR	NS	312	PCR–RFLP	Duffy negative: 2/312 Duffy positive: 310
6	Dongho et al. (2021)	Cameroon (2016–2017)	Cross-sectional study	Any age	NS	Febrile outpatients (1,001)	PCR	*SSU rRNA*	181 (37 mixed-infected with *P. falciparum*, 2 mixed-infected with *P. malariae*) (482)	PCR–RFLP	Duffy negative: 181/181
7	Fru-Cho et al. (2014)	Cameroon (2008–2009)	Cross-sectional study	18–55 years	NS	269 individuals	Microscopy, PCR	*SSU rRNA*	13 (4 mixed-infected with *P. falciparum* and *P. malariae*	PCR–RFLP, sequencing (12)	Duffy negative: 6/12 Duffy positive: 6/12
8	Gunalan et al. (2017)	Ethiopia	Cross-sectional study	NS	NS	200 symptomatic or febrile patient	Microscopy, PCR	*SSU rRNA*	200	Sequencing	Duffy negative: 2/71 Duffy positive: NA/129
9	Hamdinou et al. (2017)	Mauritania	Cross-sectional study	NS	NS	129	Microscopy, RDT	*–*	42 (129)	Indirect anti-globulin assay	Duffy negative: 16/42 Duffy positive: 26
10	Howes et al. (2018)	Madagascar (2014)	Cross-sectional study	19.6 ± 16.5	977, 47.4	2,783 eligible individuals	Microscopy, RDT and PCR	*SSU rRNA*	137 (37 mixed infected with other *Plasmodium* spp.) (275)	A microtyping kit	Duffy negative: 44/914 Duffy positive: 86/964
11	Kepple et al. (2021)	Ethiopia, Sudan	Case control study	NS	NS	305 and 107 *P. vivax* samples from Duffy-positive and Duffy-negative individuals	PCR	*SSU rRNA*	412	NS	Duffy negative: 16/107 Duffy positive: 42/305
12	Lo et al. (2015)	Ethiopia	Cross-sectional study	0–5 (72), 6–18 (128), > 18 (190)	NS	390 and 416 community and clinical samples	PCR	*SSU rRNA*	23 (73)	Sequencing	Duffy negative: 2/139 Duffy positive: 21/251
13	Lo et al. (2021)	Ethiopia, Botswana, Sudan	Cross-sectional study	NS	NS	1215 febrile patients	Microscopy, PCR	*SSU rRNA*	332	Sequencing	Duffy negative: 49/332
14	Ménard et al. (2010)	Madagascar (2007)	Cross-sectional study	3–13 years	NS	661 asymptomatic school children	Microscopy, RDT and PCR	*SSU rRNA*	128 (263)	A micro typing kit	Duffy negative: 42/476 Duffy positive: 86/185
15	Mendes et al. (2011)	Angola (2006–2007) and Equatorial Guinea (2005)	Cross-sectional study	NS	NS	995 individuals (898 from Angola and 97 from Equatorial Guinea)	PCR	*SSU rRNA*	15 (10 mixed infected with other *Plasmodium* spp.) (245)	PCR–RFLP, sequencing	Duffy negative: 15/15
16	Miri-Moghaddam et al. (2014)	Iran (2009–2012)	Case control study	Patients with *P. vivax* (29.9), patients without *P. vivax* (29.3)	NS	160 patients with *P. vivax* and 160 patients without *P. vivax* infection	Microscopy	*–*	160	PCR–RFLP, sequencing	Duffy negative: 2/6 Duffy positive: 158/314
17	Mbenda et al. (2014)	Cameroon	Cross-sectional study	1 month–82 years	104, 51.7	485 malaria symptomatic patients	PCR	*SSU rRNA*	8 (2 mixed infected with *P. falciparum*) (201)	Sequencing	Duffy negative: 8/8
18	Mbenda et al. (2016)	Cameroon	Cross-sectional study	2.3 months and 86 years	20, 33.3	60 malaria symptomatic patients	PCR	*SSU rRNA*	10 (43)	Sequencing	Duffy negative: 10/10
19	Niang et al. (2018)	Senegal (2010–2011)	Cross-sectional study	Mean 9 (8–11)	28, 58.3	48 asymptomatic school children (192 samples)	PCR	*SSU rRNA*	15 samples positive from 5 individuals (74 samples positive)	Sequencing	Duffy negative: 5/5
20	Niangaly et al. (2017)	Mali (2009–2011)	Cohort study	New born to 6 years	NS	300 children	Microscopy, PCR	*SSU rRNA*	25 (134)	Sequencing	Duffy negative: 25/25
21	Oboh et al. (2018)	Nigeria (2016–2017)	Cross-sectional study	Mean 23 (1–85)	197, 45.2	436 febrile patients (256 samples for PCR)	Microscopy, RDT and PCR	*SSU rRNA*	5 (4 mixed infected with other *Plasmodium* spp. (256)	Sequencing	Duffy negative: 5/5
22	Oboh et al. (2020)	Nigeria (2016–2017)	Cross-sectional study	25 (2–85), 26 (2–86)	109, 45	242 individuals	Microscopy, RDT and PCR	*SSU rRNA*	4 (1 mixed infected with *P. falciparum*) (145)	Sequencing	Duffy negative: 4/4
23	Poirier et al., 2016	Benin (2009–2010)	Cross-sectional study	NS	NS	1,234 Beninese blood donors (86 for PCR)	Microscopy, RDT and PCR	*SSU rRNA*	13 (86)	Sequencing	Duffy negative: 13/13
24	Russo et al. (2017)	Cameroon	Cross-sectional study	Median 24 (4–40)	191, 39.5	484 febrile outpatients	PCR	*SSU rRNA*	27 (70)	Sequencing	Duffy negative: 70/224 Duffy positive: 0/4
25	Ryan et al. (2006)	Kenya (1999–2000)	Case–control study	NS	NS	8 *P. vivax* positive cases	Microscopy, PCR	*SSU rRNA*	9 (9 mixed infected with other *Plasmodium* spp.)	flow cytometry for Fy6 and Fy3 epitopes	Duffy negative: 9/9
26	Woldearegai et al. (2013)	Ethiopia (2009)	Cross-sectional study	NS	NS	1,931 febrile patients	Microscopy, PCR	*SSU rRNA*	111 (205)	Sequencing	Duffy negative: 3/41 Duffy positive: 108/164
27	Wurtz et al. (2011)	Mauritania (2007–2009)	Cross-sectional study	NS	NS	439 febrile outpatients (277 for Duffy blood group)	PCR	Aquaglyceroporin, *P. vivax* enoylacyl carrier protein reductase, *P. ovale* P25 ookinete surface protein	110	Sequencing	Duffy negative: 1/52 Duffy positive: 109/206

*NS* Not specified.

### Study quality

Study quality was assessed using the JBI Critical Appraisal Tool (Table S2). Eighteen studies^18,19,21,23,25–29,32,34–39,41,42^ were high-quality, while nine^17,20,22,24,30,31,33,40,43^ were of moderate quality.

### The prevalence of *P. vivax* infection among Duffy-negative individuals

Twenty-seven studies^17–43^ reported *P. vivax* infection among Duffy-negative individuals. Of these, 11^17,22,30,32–38,40^ reported that all *P. vivax* infection cases were Duffy-negative (100%). These studies were conducted in West Africa (Nigeria^36,37^, Senegal^34^, Mali^35^, Benin^38^), Mid Africa (Cameroon^22,32,33^, Angola and Equatorial Guinea^30^), North Africa (Sudan^17^), and East Africa (Kenya^40^).

**Figure 3:**
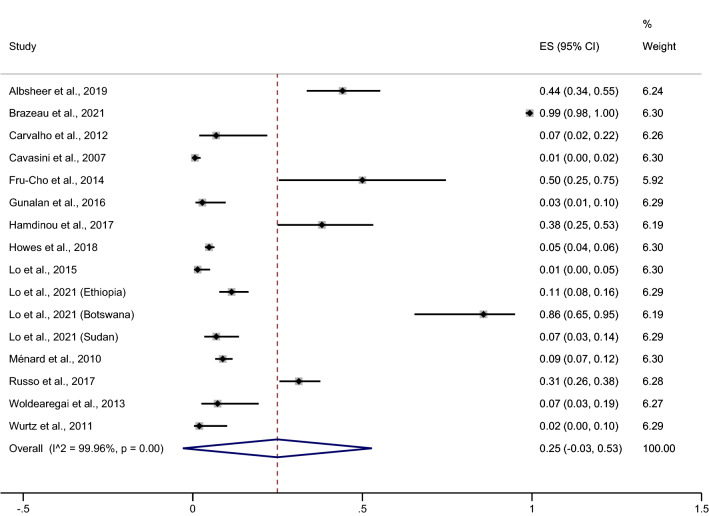
Forrest plot demonstrated the pooled prevalence of *P. vivax* infection among Duffy negative individuals. *ES* prevalence estimate, *CI* confidence interval.

Fourteen studies^18–21,23–25,27–29,39,41–43^, conducted in 16 areas and reporting *P. vivax* infection prevalence among Duffy-negative individuals, were included in the pooled prevalence meta-analysis. These results showed that the pooled prevalence was 25% (95% CI − 3%–53%, I^2^ = 99.96%, Fig. 3). Due to high heterogeneity in studies reporting this prevalence, a meta-regression analysis of the continent as a covariate was performed to test if it (the continent) was a source of heterogeneity. These results showed that the continent covariate was indeed a source of heterogeneity in the pooled prevalence (p = 0.013), therefore, further subgroup continent analyses were performed.

**Figure 4:**
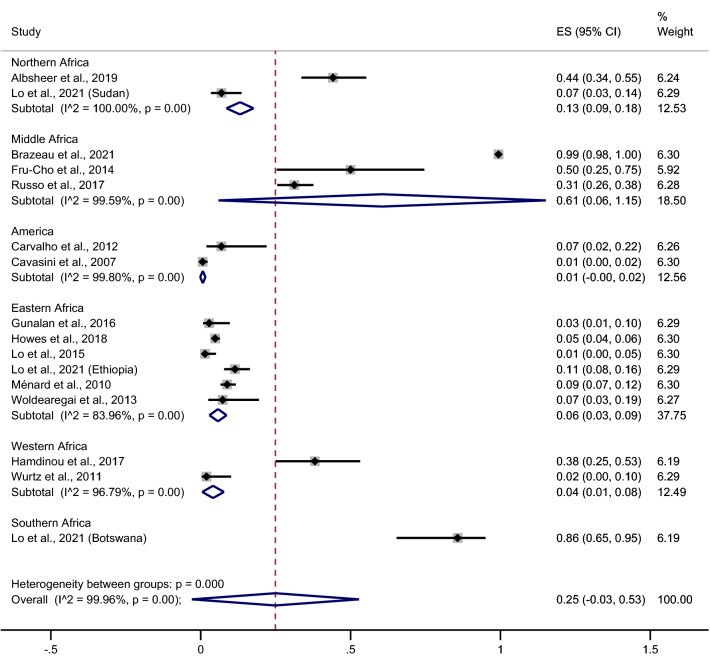
Forrest plot demonstrated the pooled prevalence of *P. vivax* infection among Duffy negative individuals stratified by continents. *ES* prevalence estimate, *CI* confidence interval.

These results indicated that the highest prevalence of *P. vivax* infection among Duffy-negative individuals was identified in a Southern African study (Botswana, 86%, 95% CI 65%–95%)^27^, followed by Mid Africa (61%, 95% CI 6%–115%, I^2^ = 99.59%, three studies ^19,23,39^), and North Africa (13%, 95% CI 9%–18%, I^2^ = 100%, two studies^18,27^). However, a low prevalence was reported in an East African study [6%, 95% CI 3%–9%, I^2^ = 83.96%, five studies (six study areas)^25,27,29,41,43^], followed by West Africa (4%, 95% CI 1%–8%, I^2^ = 96.79%, two studies^24,42^). But the lowest prevalence was reported in a South American study (Brazil, 1%, 95% CI 0%–2%, I^2^ = 99.8%, two studies^20,21^) (Fig. 4).

### The odds of *P. vivax* infection among Duffy-negative individuals

**Figure 5:**
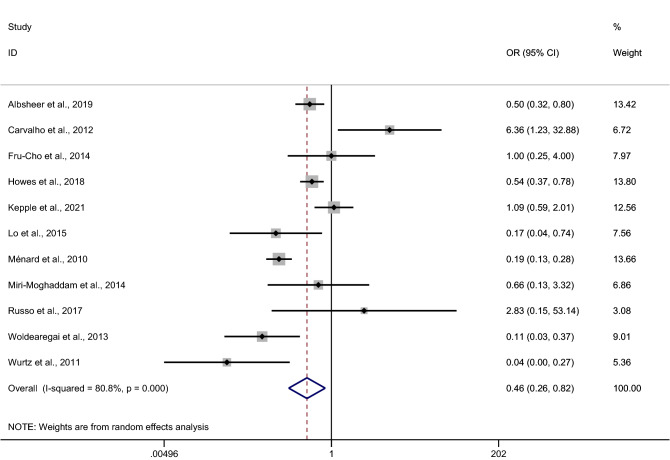
Forrest plot demonstrated the odd of *P. vivax* infection among Duffy negative individuals. *OR* odds ratio, *CI* confidence interval.

The odds of *P. vivax* infection among Duffy-negative individuals when compared with Duffy-positive individuals were estimated using data from 11 studies^12,18,20,23,25,31,39,41,44–46^. Results of individual study showed that Duffy-negativity was a protective factor for *P. vivax* infection in six studies^17,25,41,44–46^. These studies were conducted in Sudan^18^, Madagascar^25,45^, Ethiopia^41,44^, and Mauritania^46^. Only one study conducted outside Africa (Brazil) demonstrated a higher risk of *P. vivax* infection among Duffy-negative individuals^20^. No differences in infection risk were identified in four studies from Cameroon^23,39^, Ethiopia and Sudan^12^, and Iran^31^. Overall, our pooled analysis of 11 studies demonstrated a decreased odds of *P. vivax* infection among Duffy-negative individuals (p = 0.009, pooled OR 0.46, 95% CI 0.26–0.82, I^2^ = 80.8%, 11 studies, Fig. 5).

Due to a high degree of heterogeneity in some studies, a meta-regression analysis of country, continent, and study design as covariates, was performed to test if covariates were heterogeneity sources of the pooled OR; continent was identified as a heterogeneity source (p = 0.027), whereas, country and study design were not heterogeneity sources of the pooled OR (p = 0.06 and p = 0.188, respectively).

**Figure 6:**
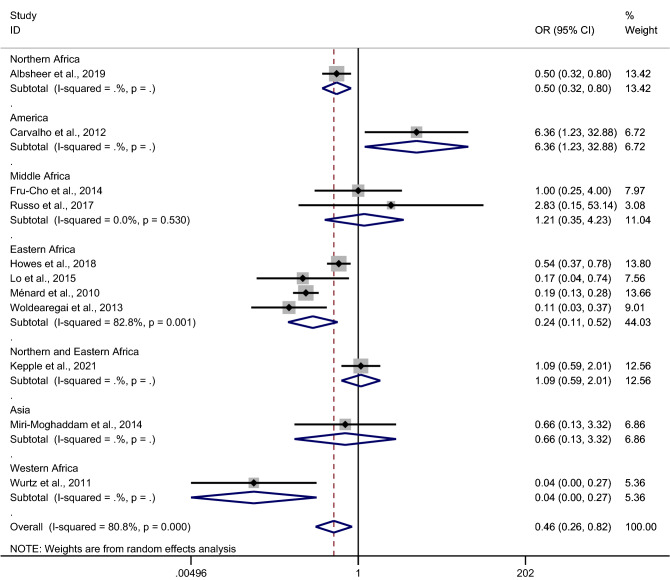
Forrest plot demonstrated the odd of *P. vivax* infection among Duffy negative individuals stratified by continents. *OR* odds ratio, *CI* confidence interval l.

Subgroup continent analysis showed that the decreased odds of *P. vivax* infection among Duffy-negative individuals were identified in studies in North Africa (OR 0.50, 95% CI 0.32–0.80)^18^, East Africa (pooled OR 0.24, 95% CI 0.11–0.52, four studies^25,28,29,41^), and West Africa (OR 0.40, 95% CI 0–0.27)^42^. Also, the increased odds of *P. vivax* infection among Duffy-negative individuals were identified in a South American study (OR 6.36, 95% CI 1.23–32.88)^20^. Other studies from Mid Africa^23,39^, North and East Africa^26^, and Asia^31^ showed no differences in the odds of infection between Duffy-negative and Duffy-positive individuals (Fig. 6).

### Sensitivity analysis

The sensitivity analysis showed that the pooled prevalence was 45% (95% CI 44%–45%, 14 studies in 16 areas, Supplementary Fig. 1). The decreased odds of infection between Duffy-negative individuals when compared with Duffy-positive individuals was p = 0.009, OR 0.46, 95% CI 0.26–0.82, 11 studies (Supplementary Fig. 2).

### Publication bias

**Figure 7:**
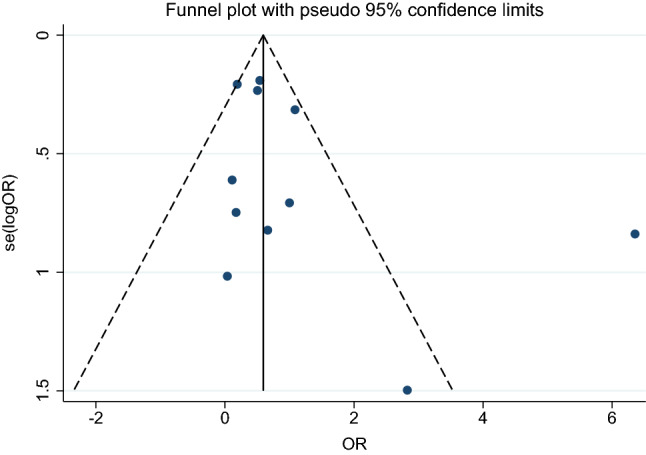
The funnel plot between odds ratio (OR) and standard error (se) of the logOR of the 11 studies demonstrated that the funnel plot was asymmetry. *OR* odds ratio, *se* standard error.

**Figure 8:**
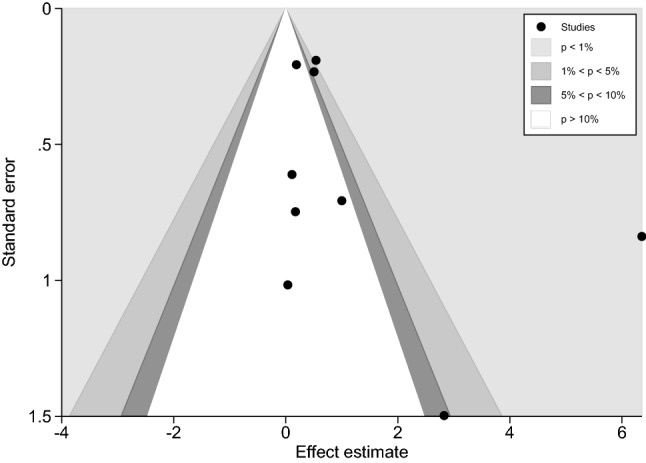
Contour-enhanced funnel plot demonstrated that the effect estimates were distributed in both significance and non-significance areas indicating that the funnel plot asymmetry was due to other causes.

A funnel plot between ES (OR) and standard error of the logES of 11 studies showed a symmetrical funnel plot (Fig. 7). Egger’s test results showed no small study effects (p = 0.188). Contour-enhanced funnel plot analyses were performed to identify if funnel plot asymmetry was due to publication bias or other causes. These results showed that the ES’s were distributed in both significant and non-significant areas, thereby suggesting funnel plot asymmetry was due to other causes (e.g., heterogeneity in the OR between studies) (Fig. 8).

## Discussion

Duffy-negative individuals are typically resistant to *P. vivax* infection; however, a recent study showed that the Duffy-negative antigen was no longer a barrier to such infections^30^. In our review, we collated 27 studies showing *P. vivax* infection among Duffy-negative individuals in Africa, including Cameroon, Ethiopia, Sudan, Botswana, Nigeria, Madagascar, Angola, Benin, Kenya, Mali, Mauritania, Democratic Republic of the Congo, and Senegal. Moreover, three studies^20,21,31^ reported infections among Duffy-negative individuals in South America (Brazil)^20,21^ and Asia (Iran)^31^.

Our qualitative analyses showed that several studies^17,22,30,32–38,40^ reported that 100% *P. vivax* infection occurred in Duffy-negative individuals. In addition, our quantitative analyses (meta-analyses) showed that the pooled prevalence of infection among Duffy-negative individuals was 25%, with a high heterogeneity across studies. These finding confirmed data from previous studies and supported the hypothesis that Duffy-negativity was no longer protective against *P. vivax* infection. Nevertheless, a high prevalence of infection among Duffy-negative individuals was observed in West Africa^34–38^), Mid Africa^19,22,23,30,32,33,39^), North Africa^17,18,27^, East Africa^40^, and Southern Africa^27^. Our meta-analysis results showed that Duffy-negativity was protective against *P. vivax* infection in individuals from East Africa^25,28,29,41^, although several reports have documented about the infection of *P. vivax* in Duffy- negative individuals. Our forest plot demonstrated the increased odds of *P. vivax* infection among Duffy-negative individuals in studies outside Africa, such as South America. This was likely caused by a low sample size, as the authors suggested *P. vivax* infections were not significantly different between Duffy-positive and Duffy-negative individuals^20^.

Several mechanisms have been postulated for *P. vivax* infections among Duffy-negative individuals. (1) Duffy-positive individuals may act as *P. vivax* reservoirs and facilitate parasite infection of Duffy-negative hepatocytes, thereby selecting new *P. vivax* strains which invade Duffy-negative erythrocytes via Duffy-independent mechanisms^45^. (2) *P. vivax* evolution for host selection may have occurred in Africa due to ideal temperatures and highly competent transmission vectors^17^. (3) In Africa, increased vector capacity to transmit other *P. vivax* malaria parasites such as *Anopheles gambiae* and *An. Arabiensis* has been observed^40,47^. Demographic factors and a high population density of young age groups may have contributed to a higher entomological inoculation rate, and contributed to *P. vivax* infection in Duffy-negative individuals, similar to *P. falciparum* infection^12,48^. (4) Parasite adaptation may have occurred for *P. knowlesi* infection rates, potentially facilitating the zoonotic transmission of specific *P. vivax* strains in Duffy-negative individuals, resulting from long exposure to *P. vivax* infections in African populations. In studies on simian malaria parasites requiring the Duffy protein antigen for erythrocyte invasion, *P. knowlesi* invaded Duffy-negative erythrocytes, suggesting a Duffy-independent *P. knowlesi* infection mechanism^49^. (5) *P. vivax* can hide in the bone marrow of Duffy-negative hosts and persist as low parasitemic, asymptomatic infections^50^. (6) Difference in latitude in some areas could affect *P. vivax* transmission, e.g., higher altitudes in Cameroon^11^, therefore, *P. vivax* could infect populations in these areas rather than *P. falciparum,* suggesting *P. vivax* abilities to infect populations in higher altitudes^51^. (7) *P. vivax* may use several receptor-ligand interactions to tightly bind erythrocytes in the absence of a Duffy receptor, e.g., the glycophosphatidylinositol-anchored micronemal antigen or tryptophan-rich antigens^52^.

Our study had some limitations. Firstly, we identified a limited number of studies reporting *P. vivax* infection among Duffy-negative individuals. Secondly, we identified high heterogeneity among studies. Thirdly, we observed funnel plot asymmetry which was likely caused by heterogeneity of the ES among studies. Although subgroup analyses were performed, the heterogeneity persisted. Therefore, our results must be interpreted with caution.

## Conclusions

Our systematic review and meta-analysis confirmed that *P. vivax* infected Duffy-negative individuals over a wide prevalence range from 0 to 100% depending on different geographical areas. Future investigations are required to determine if Duffy-negativity is still protective for *P. vivax* infection.

## Supplementary Information

Supplementary Legends.

Supplementary Figure 1.

Supplementary Figure 2.

Supplementary Table S1.

Supplementary Table S2.

## Data Availability

All data related to this study are available in this manuscript.

## References

[1] World Malaria Report 2019. https://www.who.int/publications/i/item/9789241565721.

[2] Twohig KA, Pfeffer DA, Baird JK, Price RN, Zimmerman PA, Hay SI, Gething PW, Battle KE, Howes RE (2019). Growing evidence of *Plasmodium vivax* across malaria-endemic Africa. PLoS Negl. Trop. Dis..

[3] Oboh MA, Oyebola KM, Idowub ET, Badianea AS, Otubanjo OA, Ndiayea D (2020). Rising report of *Plasmodium vivax* in sub-Saharan Africa: Implications for malaria elimination agenda. Sci. Afr..

[4] Miller LH, Mason SJ, Clyde DF, McGinniss MH (1976). The resistance factor to *Plasmodium vivax* in blacks: The Duffy-blood-group genotype, FyFy. N. Engl. J. Med..

[5] Howes RE, Patil AP, Piel FB, Nyangiri OA, Kabaria CW, Gething PW, Zimmerman PA, Barnadas C, Beall CM, Gebremedhin A (2011). The global distribution of the Duffy blood group. Nat. Commun..

[6] Chaudhuri A, Zbrzezna V, Johnson C, Nichols M, Rubinstein P, Marsh WL, Pogo AO (1989). Purification and characterization of an erythrocyte membrane protein complex carrying Duffy blood group antigenicity. Possible receptor for *Plasmodium vivax* and *Plasmodium knowlesi* malaria parasite. J. Biol. Chem..

[7] Chitnis CE, Sharma A (2008). Targeting the *Plasmodium vivax* Duffy-binding protein. Trends Parasitol..

[8] Chaudhuri A, Polyakova J, Zbrzezna V, Williams K, Gulati S, Pogo AO (1993). Cloning of glycoprotein D cDNA, which encodes the major subunit of the Duffy blood group system and the receptor for the *Plasmodium vivax* malaria parasite. Proc. Natl. Acad. Sci. USA.

[9] King CL, Adams JH, Xianli J, Grimberg BT, McHenry AM, Greenberg LJ, Siddiqui A, Howes RE, da Silva-Nunes M, Ferreira MU, Zimmerman PA (2011). Fy(a)/Fy(b) antigen polymorphism in human erythrocyte Duffy antigen affects susceptibility to *Plasmodium vivax* malaria. Proc. Natl. Acad. Sci. USA.

[10] Hoher G, Fiegenbaum M, Almeida S (2018). Molecular basis of the Duffy blood group system. Blood Transfus.

[11] Djeunang Dongho GB, Gunalan K, L'Episcopia M, Paganotti GM, Menegon M, Sangong RE, Georges BM, Fondop J, Severini C, Sobze MS (2021). Plasmodium vivax infections detected in a large number of febrile Duffy-negative Africans in Dschang, Cameroon. Am. J. Trop. Med. Hyg..

[12] Kepple D, Hubbard A, Ali MM, Abargero BR, Lopez K, Pestana K, Janies DA, Yan G, Hamid MM, Yewhalaw D, Lo E (2021). Plasmodium vivax from Duffy-negative and Duffy-positive individuals shares similar gene pool in east Africa. J. Infect. Dis..

[13] Gunalan K, Niangaly A, Thera MA, Doumbo OK, Miller LH (2018). *Plasmodium vivax* infections of Duffy-negative erythrocytes: Historically undetected or a recent adaptation?. Trends Parasitol..

[14] Kotepui M, Kotepui KU, Milanez GJ, Masangkay FR (2020). Prevalence and risk factors related to poor outcome of patients with severe *Plasmodium vivax* infection: A systematic review, meta-analysis, and analysis of case reports. BMC Infect. Dis..

[15] Moher D, Liberati A, Tetzlaff J, Altman DG, Group P (2009). Preferred reporting items for systematic reviews and meta-analyses: The PRISMA statement. PLoS Med..

[16] Moola SMZ, Tufanaru C, Aromataris E, Sears K, Sfetcu R, Currie M, Qureshi R, Mattis P, Lisy K, Mu P-F (2020). Systematic Reviews of Etiology and Risk.

[17] Abdelraheem MH, Albsheer MM, Mohamed HS, Amin M, Abdel Hamid MM (2016). Transmission of *Plasmodium vivax* in Duffy-negative individuals in central Sudan. Trans. R. Soc. Trop. Med. Hyg..

[18] Albsheer MMA, Pestana K, Ahmed S, Elfaki M, Gamil E, Ahmed SM, Ibrahim ME, Musa AM, Lo E, Abdel Hamid MM (2019). Distribution of duffy phenotypes among *Plasmodium vivax* infections in Sudan. Genes.

[19] Brazeau NF, Mitchell CL, Morgan AP, Deutsch-Feldman M, Watson OJ, Thwai KL, Gelabert P, van Dorp L, Keeler CY, Waltmann A (2021). The epidemiology of *Plasmodium vivax* among adults in the Democratic Republic of the Congo. Nat. Commun..

[20] Carvalho TA, Queiroz MG, Cardoso GL, Diniz IG, Silva AN, Pinto AY, Guerreiro JF (2012). *Plasmodium vivax* infection in Anajás, State of Pará: No differential resistance profile among Duffy-negative and Duffy-positive individuals. Malar. J..

[21] Cavasini CE, de Mattos LC, D'Almeida Couto AAR, D'Almeida Couto VSC, Gollino Y, Moretti LJ, Bonini-Domingos CR, Rossit ARB, Castilho L, Machado RLD (2007). Duffy blood group gene polymorphisms among malaria vivax patients in four areas of the Brazilian Amazon region. Malar. J..

[22] Djeunang Dongho GB, Gunalan K, L'Episcopia M, Paganotti GM, Menegon M, Efeutmecheh Sangong R, Bouting Mayaka G, Fondop J, Severini C, Sanou Sobze M (2021). *Plasmodium vivax* infections detected in a large number of febrile Duffy-negative Africans in Dschang, Cameroon. Am. J. Trop. Med. Hyg..

[23] Fru-Cho J, Bumah VV, Safeukui I, Nkuo-Akenji T, Titanji VP, Haldar K (2014). Molecular typing reveals substantial *Plasmodium vivax* infection in asymptomatic adults in a rural area of Cameroon. Malar. J..

[24] Hamdinou MM, Deida J, Ebou MH, El-Ghassem A, Lekweiry KM, Salem M, Tahar R, Simard F, Basco L, Boukhary A (2017). Distribution of Duffy blood group (FY) phenotypes among *Plasmodium vivax*-infected patients in Nouakchott, Mauritania. Trop. Med. Int. Health.

[25] Howes RE, Franchard T, Rakotomanga TA, Ramiranirina B, Zikursh M, Cramer EY, Tisch DJ, Kang SY, Ramboarina S, Ratsimbasoa A, Zimmerman PA (2018). Risk factors for malaria infection in central Madagascar: Insights from a cross-sectional population survey. Am. J. Trop. Med. Hyg..

[26] Kepple D, Hubbard A, Ali MM, Abargero BR, Lopez K, Pestana K, Janies DA, Yan GY, Hamid MM, Yewhalaw D, Lo E (2021). *Plasmodium vivax* from Duffy-negative and Duffy-positive individuals share similar gene pools in east Africa. J. Infect. Dis..

[27] Lo E, Russo G, Pestana K, Kepple D, Abagero BR, Dongho GBD, Gunalan K, Miller LH, Hamid MMA, Yewhalaw D, Paganotti GM (2021). Contrasting epidemiology and genetic variation of *Plasmodium vivax* infecting Duffy-negative individuals across Africa. Int. J. Infect. Dis..

[28] Lo E, Yewhalaw D, Zhong DB, Zemene E, Degefa T, Tushune K, Ha M, Lee MC, James AA, Yan GY (2015). Molecular epidemiology of *Plasmodium vivax* and *Plasmodium falciparum* malaria among Duffy-positive and Duffy-negative populations in Ethiopia. Malar. J..

[29] Ménard D, Barnadas C, Bouchier C, Henry-Halldin C, Gray LR, Ratsimbasoa A, Thonier V, Carod JF, Domarle O, Colin Y (2010). *Plasmodium vivax* clinical malaria is commonly observed in Duffy-negative Malagasy people. Proc. Natl. Acad. Sci. USA.

[30] Mendes C, Dias F, Figueiredo J, Mora VG, Cano J, de Sousa B, de Rosario VE, Benito A, Berzosa P, Arez AP (2011). Duffy negative antigen is no longer a barrier to *Plasmodium vivax*: Molecular evidences from the African West Coast (Angola and Equatorial Guinea**)**. PLoS Negl. Trop. Dis..

[31] Miri-Moghaddam E, Bameri Z, Mohamadi M (2014). Duffy blood group genotypes among malaria *Plasmodium vivax* patients of Baoulch population in Southeastern Iran. Asian Pac. J. Trop. Med..

[32] NgassaMbenda HG, Das A (2014). Molecular evidence of *Plasmodium vivax* mono and mixed malaria parasite infections in Duffy-negative native Cameroonians. PLoS ONE.

[33] Ngassa Mbenda HG, Gouado I, Das A (2016). An additional observation of *Plasmodium vivax* malaria infection in Duffy-negative individuals from Cameroon. J. Infect. Dev. Ctries.

[34] Niang M, Sane R, Sow A, Sadio BD, Chy S, Legrand E, Faye O, Diallo M, Sall AA, Menard D, Toure-Balde A (2018). Asymptomatic *Plasmodium vivax* infections among Duffy-negative population in Kedougou, Senegal. Trop. Med. Health.

[35] Niangaly A, Karthigayan G, Amed O, Coulibaly D, Sá JM, Adams M, Travassos MA, Ferrero J, Laurens MB, Kone AK (2017). *Plasmodium vivax* infections over 3 years in Duffy blood group negative Malians in Bandiagara, Mali. Am. J. Trop. Med. Hyg..

[36] Oboh MA, Badiane AS, Ntadom G, Ndiaye YD, Diongue K, Diallo MA, Ndiaye D (2018). Molecular identification of *Plasmodium* species responsible for malaria reveals *Plasmodium vivax* isolates in Duffy negative individuals from southwestern Nigeria. Malar. J..

[37] Oboh MA, Singh US, Singh US, Ndiaye D, Badiane AS, Ali NA, Bharti PK, Das A (2020). Presence of additional *Plasmodium vivax* malaria in Duffy negative individuals from Southwestern Nigeria. Malar. J..

[38] Poirier P, Doderer-Lang C, Atchade PS, Lemoine JP, de l’Isle MC, Abou-Bacar A, Pfaff AW, Brunet J, Arnoux L, Haar E (2016). The hide and seek of *Plasmodium vivax* in West Africa: Report from a large-scale study in Beninese asymptomatic subjects. Malar. J..

[39] Russo G, Faggioni G, Paganotti GM, Djeunang Dongho GB, Pomponi A, De Santis R, Tebano G, Mbida M, Sanou Sobze M, Vullo V (2017). Molecular evidence of *Plasmodium vivax* infection in Duffy negative symptomatic individuals from Dschang, West Cameroon. Malar. J..

[40] Ryan JR, Stoute JA, Amon J, Dunton RF, Mtalib R, Koros J, Owour B, Luckhart S, Wirtz RA, Barnwell JW, Rosenberg R (2006). Evidence for transmission of *Plasmodium vivax* among a duffy antigen negative population in Western Kenya. Am. J. Trop. Med. Hyg..

[41] Woldearegai TG, Kremsner PG, Kun JF, Mordmüller B (2013). *Plasmodium vivax* malaria in Duffy-negative individuals from Ethiopia. Trans. R. Soc. Trop. Med. Hyg..

[42] Wurtz N, Mint Lekweiry K, Bogreau H, Pradines B, Rogier C, Ould Mohamed Salem Boukhary A, Hafid JE, Ould Ahmedou Salem MS, Trape JF, Basco LK, Briolant S (2011). Vivax malaria in Mauritania includes infection of a Duffy-negative individual. Malar. J..

[43] Gunalan K, Lo E, Hostetler JB, Yewhalaw D, Mu J, Neafsey DE, Yan G, Miller LH (2016). Role of *Plasmodium vivax* Duffy-binding protein 1 in invasion of Duffy-null Africans. Proc. Natl. Acad. Sci. USA.

[44] Lo E, Yewhalaw D, Zhong D, Zemene E, Degefa T, Tushune K, Ha M, Lee MC, James AA, Yan G (2015). Molecular epidemiology of *Plasmodium vivax* and *Plasmodium falciparum* malaria among Duffy-positive and Duffy-negative populations in Ethiopia. Malar. J.

[45] Menard D, Barnadas C, Bouchier C, Henry-Halldin C, Gray LR, Ratsimbasoa A, Thonier V, Carod JF, Domarle O, Colin Y (2010). *Plasmodium vivax* clinical malaria is commonly observed in Duffy-negative Malagasy people. Proc. Natl. Acad. Sci. USA.

[46] Wurtz N, Lekweiry KM, Bogreau H, Pradines B, Rogier C, Boukhary A, Hafid JE, Salem M, Trape JF, Basco LK, Briolant S (2011). Vivax malaria in Mauritania includes infection of a Duffy-negative individual. Malar. J..

[47] Taye A, Hadis M, Adugna N, Tilahun D, Wirtz RA (2006). Biting behavior and *Plasmodium* infection rates of *Anopheles arabiensis* from Sille, Ethiopia. Acta Trop.

[48] Vafa M, Troye-Blomberg M, Anchang J, Garcia A, Migot-Nabias F (2008). Multiplicity of *Plasmodium falciparum* infection in asymptomatic children in Senegal: relation to transmission, age and erythrocyte variants. Malar. J..

[49] Mason SJ, Miller LH, Shiroishi T, Dvorak JA, McGinniss MH (1977). The Duffy blood group determinants: their role in the susceptibility of human and animal erythrocytes to *Plasmodium knowlesi* malaria. Br. J. Haematol..

[50] Obaldia N, Meibalan E, Sa JM, Ma S, Clark MA, Mejia P, Moraes Barros RR, Otero W, Ferreira MU, Mitchell JR (2018). Bone marrow is a major parasite reservoir in *Plasmodium vivax* infection. MBio.

[51] Bango ZA, Tawe L, Muthoga CW, Paganotti GM (2020). Past and current biological factors affecting malaria in the low transmission setting of Botswana: A review. Infect. Genet. Evol..

[52] Chan LJ, Dietrich MH, Nguitragool W, Tham WH (2020). *Plasmodium vivax* reticulocyte binding proteins for invasion into reticulocytes. Cell Microbiol..

