# Supplementary material for: Prevalence and risk of Plasmodium vivax infection among Duffy-negative individuals: a systematic review and meta-analysis
Source: Sci Rep. 2022 Mar 7;12:3998. doi: 10.1038/s41598-022-07711-5 (PMC8901689; doi:10.1038/s41598-022-07711-5)
Supplement: Supplementary file 1 — Supplementary Legends. [file 41598_2022_7711_MOESM1_ESM.docx]

Supplementary Figure 1. The pooled prevalence of *P. vivax* infection between Duffy negative individuals using the fixed effects model.

Supplementary Figure 2. The odds of *P. vivax* infection among Duffy negative individuals compared to Duffy positive individuals using the fixed effects model.
