# Supplementary figures and images for: Prevalence and risk of Plasmodium vivax infection among Duffy-negative individuals: a systematic review and meta-analysis
Source: Sci Rep. 2022 Mar 7;12:3998. doi: 10.1038/s41598-022-07711-5 (PMC8901689; doi:10.1038/s41598-022-07711-5)

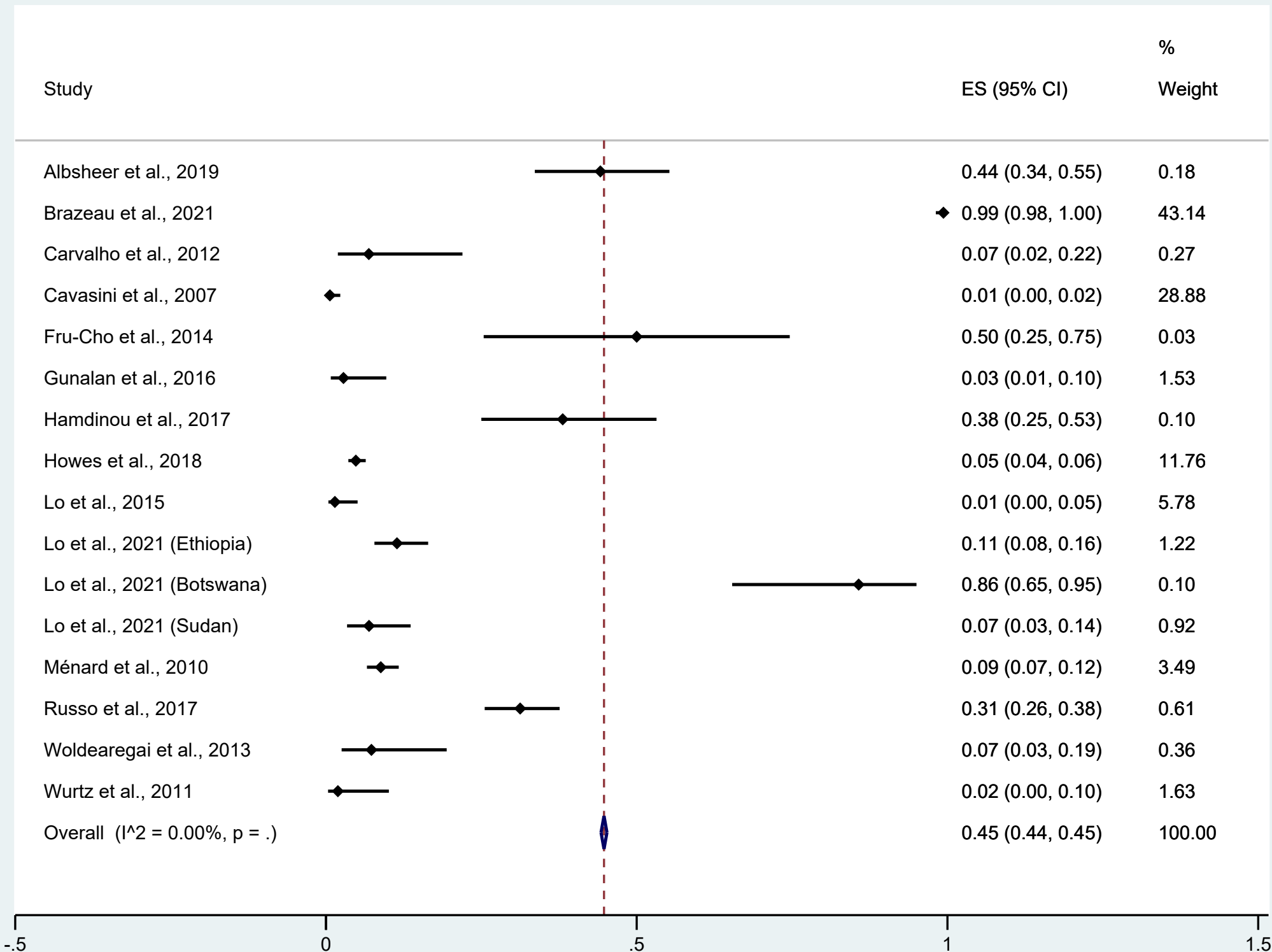

Supplement: Supplementary file 2 — Supplementary Figure 1. [file 41598_2022_7711_MOESM2_ESM.pdf]

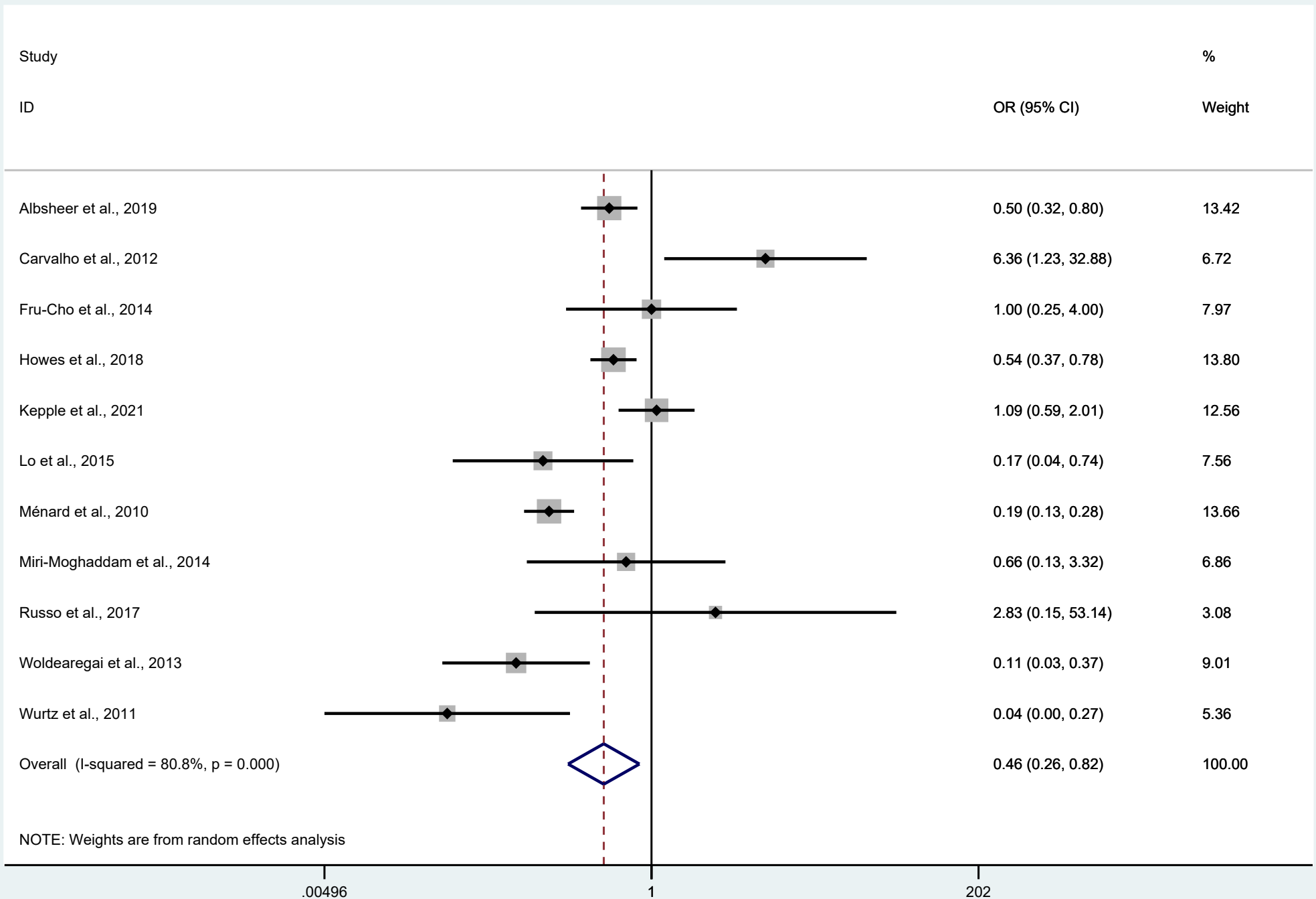

Supplement: Supplementary file 3 — Supplementary Figure 2. [file 41598_2022_7711_MOESM3_ESM.pdf]
