# Supplementary material for: Prevalence and risk of Plasmodium vivax infection among Duffy-negative individuals: a systematic review and meta-analysis
Source: Sci Rep. 2022 Mar 7;12:3998. doi: 10.1038/s41598-022-07711-5 (PMC8901689; doi:10.1038/s41598-022-07711-5)
Supplement: Supplementary file 4 — Supplementary Table S1. [file 41598_2022_7711_MOESM4_ESM.docx]

**Prevalence and risk of *Plasmodium vivax* infection among Duffy-negative individuals: a systematic review and meta-analysis**

Polrat Wilairatana^1^, Frederick Ramirez Masangkay^2^, Kwuntida Uthaisar Kotepui ^3^, Giovanni De Jesus Milanez^4^, Manas Kotepui^3*^

^1^Department of Clinical Tropical Medicine, Faculty of Tropical Medicine, Mahidol University, Bangkok, Thailand

^2^Department of Medical Technology, Institute of Arts and Sciences, Far Eastern University-Manila, Manila, Philippines

^3^Medical Technology, School of Allied Health Sciences, Walailak University, Tha Sala, Nakhon Si Thammarat, Thailand

^4^Department of Medical Technology, Faculty of Pharmacy, University of Santo Tomas, Manila, Philippines.

Authors’ e-mail addresses:

**^*^Corresponding Author**: Manas Kotepui; manas.ko@wu.ac.th, manaskote@gmail.com

Polrat Wilairatana; polrat.wil@mahidol.ac.th

Frederick Ramirez Masangkay; frederick_masangkay2002@yahoo.com

Kwuntida Uthaisar Kotepui; kwuntida.ut@wu.ac.th

Giovanni De Jesus Milanez; gmilanez81@gmail.com

**S1 Table. Search term**

| **Databases** | **Search terms** | **Search date** |
| --- | --- | --- |
| MEDLINE via PubMed | (DARC OR Duffy OR "Fy glycoprotein" OR "FY protein" OR "DARC antigen" OR "Duffy antigen-chemokine receptor" OR "DARC protein" OR ACKR1 OR DBP OR “D binding protein” OR “D-element-binding protein” OR “DBP transcription factor” OR “D-site binding protein”) AND vivax | 22 February 2021 to 31 January 2022 |
| Scopus | (DARC OR Duffy OR "Fy glycoprotein" OR "FY protein" OR "DARC antigen" OR "Duffy antigen-chemokine receptor" OR "DARC protein" OR ACKR1 OR DBP OR “D binding protein” OR “D-element-binding protein” OR “DBP transcription factor” OR “D-site binding protein”) AND vivax | 22 February 2021 to 31 January 2022 |
| Web of Science | (DARC OR Duffy OR "Fy glycoprotein" OR "FY protein" OR "DARC antigen" OR "Duffy antigen-chemokine receptor" OR "DARC protein" OR ACKR1 OR DBP OR “D binding protein” OR “D-element-binding protein” OR “DBP transcription factor” OR “D-site binding protein”) AND vivax | 22 February 2021 to 31 January 2022 |
