# Supplementary material for: Prevalence and risk of Plasmodium vivax infection among Duffy-negative individuals: a systematic review and meta-analysis
Source: Sci Rep. 2022 Mar 7;12:3998. doi: 10.1038/s41598-022-07711-5 (PMC8901689; doi:10.1038/s41598-022-07711-5)
Supplement: Supplementary file 5 — Supplementary Table S2. [file 41598_2022_7711_MOESM5_ESM.docx]

**Prevalence and risk of *Plasmodium vivax* infection among Duffy-negative individuals: a systematic review and meta-analysis**

Polrat Wilairatana^1^, Frederick Ramirez Masangkay^2^, Kwuntida Uthaisar Kotepui ^3^, Giovanni De Jesus Milanez^4^, Manas Kotepui^3*^

^1^Department of Clinical Tropical Medicine, Faculty of Tropical Medicine, Mahidol University, Bangkok, Thailand

^2^Department of Medical Technology, Institute of Arts and Sciences, Far Eastern University-Manila, Manila, Philippines

^3^Medical Technology, School of Allied Health Sciences, Walailak University, Tha Sala, Nakhon Si Thammarat, Thailand

^4^Department of Medical Technology, Faculty of Pharmacy, University of Santo Tomas, Manila, Philippines.

Authors’ e-mail addresses:

**^*^Corresponding Author**: Manas Kotepui; manas.ko@wu.ac.th, manaskote@gmail.com

Polrat Wilairatana; polrat.wil@mahidol.ac.th

Frederick Ramirez Masangkay; frederick_masangkay2002@yahoo.com

Kwuntida Uthaisar Kotepui; kwuntida.ut@wu.ac.th

Giovanni De Jesus Milanez; gmilanez81@gmail.com

Cross-sectional studies

| No. | Authors | Eligibility criteria | Study subjects and the setting | Exposure measured in a valid and reliable way 'gold standard' | A specified diagnosis or definition | Identification of confounding factors | Strategy to dealing with confounding factors | Outcomes measured in a valid and reliable way | Appropriate statistical analysis | Scores (8) | Quality (low, moderate, high) |
| --- | --- | --- | --- | --- | --- | --- | --- | --- | --- | --- | --- |
| 1 | Abdelraheem et al., 2016 | Yes | Yes | Yes | Yes | No | No | Yes | Yes | 6 | Moderate |
| 2 | Albsheer et al., 2019 | Yes | Yes | Yes | Yes | Yes | Yes | Yes | Yes | 8 | High |
| 3 | Brazeau et al., 2021 | Yes | Yes | Yes | Yes | Yes | Yes | Yes | Yes | 8 | High |
| 4 | Carvalho et al., 2012 | Yes | Yes | Yes | Yes | No | No | Yes | Yes | 6 | Moderate |
| 5 | Cavasini et al., 2007 | Yes | Yes | Yes | Yes | Yes | Yes | Yes | Yes | 8 | High |
| 6 | Dongho et al., 2021 | Yes | Yes | Yes | Yes | No | No | Yes | Yes | 6 | Moderate |
| 7 | Fru-Cho et al., 2014 | Yes | Yes | Yes | Yes | Yes | Yes | Yes | Yes | 8 | High |
| 8 | Gunalan et al., 2017 | No | Yes | Yes | Yes | No | No | Yes | Yes | 5 | Moderate |
| 9 | Hamdinou et al., 2017 | No | No | Yes | Yes | No | No | Yes | Yes | 5 | Moderate |
| 10 | Howes et al., 2018 | Yes | Yes | Yes | Yes | Yes | Yes | Yes | Yes | 8 | High |
| 11 | Lo et al., 2015 | Yes | Yes | Yes | Yes | Yes | Yes | Yes | Yes | 8 | High |
| 12 | Lo et al., 2021 | Yes | Yes | Yes | Yes | Yes | Yes | Yes | Yes | 8 | High |
| 13 | Ménard et al., 2010 | Yes | Yes | Yes | Yes | Yes | Yes | Yes | Yes | 8 | High |
| 14 | Mendes et al., 2011 | Yes | Yes | Yes | Yes | No | No | Yes | Yes | 6 | Moderate |
| 15 | Mbenda et al., 2014 | Yes | Yes | Yes | Yes | Yes | Yes | Yes | Yes | 8 | High |
| 16 | Mbenda et al., 2016 | Yes | Yes | Yes | Yes | No | No | Yes | Yes | 6 | Moderate |
| 17 | Niang et al., 2018 | Yes | Yes | Yes | Yes | Yes | Yes | Yes | Yes | 8 | High |
| 18 | Oboh et al., 2018 | Yes | Yes | Yes | Yes | Yes | Yes | Yes | Yes | 8 | High |
| 19 | Oboh et al., 2020 | Yes | Yes | Yes | Yes | No | Yes | Yes | Yes | 7 | High |
| 20 | Poirier et al., 2016 | Yes | Yes | Yes | Yes | Yes | Yes | Yes | Yes | 8 | High |
| 21 | Russo et al., 2017 | Yes | Yes | Yes | Yes | Yes | Yes | Yes | Yes | 8 | High |
| 22 | Woldearegai et al., 2013 | Yes | Yes | Yes | Yes | Yes | Yes | Yes | Yes | 8 | High |
| 23 | Wurtz et al., 2011 | Yes | Yes | Yes | Yes | Yes | Yes | Yes | Yes | 8 | High |

NA, Not Applicable

Case-control studies

| No. | Authors | Comparable of the group | Appropriate of matching case and control groups | Same criteria used for identification of cases and controls | Exposure measured in a valid and reliable way 'gold standard' | Exposure measured in the same way for cases and controls | Confounding factors | Dealing with confounding factors | Outcomes measured in a valid and reliable way | Exposure period of interest long enough to be meaningful | Appropriate statistical analysis | Scores (10) | Quality (low, moderate, high) |
| --- | --- | --- | --- | --- | --- | --- | --- | --- | --- | --- | --- | --- | --- |
| 1 | Kepple et al., 2021 | Yes | Yes | Yes | Yes | Yes | Yes | Yes | Yes | Yes | Yes | 10 | High |
| 2 | Miri-Moghaddam et al., 2014 | Yes | Yes | Yes | Yes | Yes | No | No | Yes | No | Yes | 7 | Moderate |
| 3 | Ryan et al., 2006 | Yes | Yes | Yes | Yes | Yes | No | No | Yes | No | No | 6 | Moderate |

NA, Not Applicable

Cohort study

| No. | Authors | The two groups similar and recruited from the same population | The exposures measured similarly to assign people to both exposed and unexposed groups | Exposure measured in a valid and reliable way 'gold standard' | Confounding factors | Dealing with confounding factors | The groups/participants free of the outcome at the start of the study | The outcomes measured in a valid and reliable way | The follow up time reported and sufficient to be long enough for outcomes to occur | Was follow up complete, and if not, were the reasons to loss to follow up described and explored | Strategies to address incomplete follow up utilized | Appropriate statistical analysis | Scores (11) | Quality (low, moderate, high) |
| --- | --- | --- | --- | --- | --- | --- | --- | --- | --- | --- | --- | --- | --- | --- |
| 1 | Niangaly et al., 2017 | Yes | Yes | Yes | Yes | Yes | Yes | Yes | Yes | Yes | Yes | Yes | 11 | High |

NA, Not Applicable
